# Shigella sonnei and Shigella flexneri infection in Caenorhabditis elegans led to species-specific regulatory responses in the host and pathogen

**DOI:** 10.1099/mgen.0.001339

**Published:** 2025-01-24

**Authors:** Bao Chi Wong, Hock Siew Tan

**Affiliations:** 1 School of Science, Monash University Malaysia, 47500 Bandar Sunway, Selangor Darul Ehsan, Subang Jaya, Malaysia

**Keywords:** *Caenorhabditis elegans*, dual-RNA sequencing, infection, *Shigella flexneri*, *Shigella sonnei*

## Abstract

In recent decades, *Shigella sonnei* has surpassed *Shigella flexneri* as the leading cause of shigellosis, possibly due to species-specific differences in their transcriptomic responses. This study used dual RNA sequencing to analyse the transcriptomic responses of *Caenorhabditis elegans* and the two *Shigella* species at early (10 minutes) and late (24 hours) stages of infection. While the nematode defence response was downregulated during both *Shigella* infections, only infection by *S. sonnei* led to downregulation of sphingolipid metabolism, cadmium ion response and xenobiotic response in *C. elegans*. Furthermore, *S. sonnei* upregulates biofilm formation and energy generation/conservation during infection, acid resistance-related genes and biofilm regulators compared to *S. flexneri*. These findings highlight species-specific responses during *C. elegans* infection.

**Impact Statement**

Shigellosis, a gastrointestinal infection predominantly caused by *Shigella sonnei* or *Shigella flexneri*, severely affects vulnerable populations, particularly young children. While *S. flexneri* is predominantly isolated in developing countries and *S. sonnei* predominantly in industrialized countries, recent decades have seen a shift from *S. flexneri* to *S. sonnei* predominance in developing countries. Unfortunately, the reasons remain unclear. To elucidate this shift, this study investigates species-specific transcriptomic responses during *Caenorhabditis elegans* infection. We found that during *C. elegans* infection, *S. sonnei* and *S. flexneri* elicit distinct responses in *C. elegans*, where only *S. sonnei* led to the downregulation of sphingolipid metabolism in the nematodes. Additionally, *S. sonnei* upregulates genes related to biofilm formation and acid resistance during infection as compared to *S. flexneri* under similar conditions. These findings provide critical insights into the etiological shift and significantly contribute to the literature on shigellosis pathogenesis.

## Data Availability

Trimmed paired-end data of the dual RNA sequencing (*Caenorhabditis elegans–Shigella sonnei* and *Caenorhabditis elegans*–*Shigella flexneri*) are available from the Sequencing Read Archive under BioProject PRJNA1128419 [10 minutes post-infection (mpi)] and PRJNA1147013 [24 hours post-infection (hpi)]. Raw sequencing data of *S. sonnei* on nematode growth medium (NGM) agar (*in* vitro) are available under BioProject PRJNA1148041. Other datasets used and analysed during the current study are available from the corresponding author upon reasonable request.

## Introduction

*Shigella* is the bacterial species responsible for causing shigellosis, an intestinal infection associated with aggressive and bloody diarrhoea [1]. Among the four species of *Shigella*, *Shigella flexneri* is associated with resource-poor countries, while *Shigella sonnei* is associated with developed countries [2,3]. As more countries undergo significant industrialization, there is an increase in the proportion of *S. sonnei* isolates compared to *S. flexneri* [3,8]. Much information on the virulence and pathogenesis of *Shigella* was obtained from *S. flexneri* studies and generalized to other *Shigella* species. However, recent studies have demonstrated the species-specific differences between *S. flexneri* and *S. sonnei*. For instance, it is commonly reported that *S. flexneri* uses the type III secretion system (T3SS) during macrophage internalization [9,10]. However, *S. sonnei*’s internalization into macrophages is independent of T3SS [11]. The O-antigen of *S. sonnei* is also different from other *Shigella* [12], which helps decrease macrophage internalization and pyroptosis [11] and increases virulence in the zebrafish model [13]. Other than that, *S. sonnei* also uses colicins to outcompete other bacteria during infection [14,15]. These studies demonstrated that *S. sonnei* studies are necessary due to the species-specific differences from *S. flexneri*.

*Caenorhabditis elegans* is a soil-dwelling nematode that has been used as an animal model for bacterial infection for multiple bacterial species including *Pseudomonas aeruginosa* [16,17], *Staphylococcus aureus* [18,19] and *Shigella* [20,23]. The intestinal epithelial cells of the nematodes are similar to human intestinal cells, making them a valuable *in vivo* model for preliminary studies of host–pathogen interactions [24,25]. In contrast, multiple studies were performed on the *Shigella*–*C. elegans* model, and they were focused on the host response towards *Shigella* infection [21,23, 26, 27]. To our knowledge, this study is the first to analyse the *Shigella* response during *C. elegans* infection.

This study aimed to investigate both the host response and the pathogen response during the infection through dual RNA sequencing, where the transcriptomic data of both organisms are analysed during the bacterial infection. This study demonstrated that the infection by *S. sonnei* and *S. flexneri* can lead to different responses in the *C. elegans* host at early (10 minutes post-infection, or 10 mpi) and later (24 hours post-infection, or 24 hpi) stages of infection. The two *Shigella* also had different responses during *C. elegans* infection.

## Methodology

### Strains, plasmids and growth conditions

The *C. elegans* wild-type N2 Bristol strain was cultured on nematode growth medium (NGM) agar plates seeded with UV-killed *Escherichia coli* OP50 and maintained at 22 °C [28]. *E. coli* OP50 was used as the standardized food source for *C. elegans* in laboratory conditions and is therefore non-pathogenic [28]. *S. sonnei* ATCC 29930 and *S. flexneri* ATCC 12022 were obtained from the American Type Culture Collection (ATCC), and they are pathogenic towards *C. elegans*. Unless stated otherwise, all bacterial strains were grown on Luria-Bertani agar and broth (Himedia, India) and incubated overnight at 37 °C.

### Exposure of *C. elegans* to bacterial strains, RNA extraction and sequencing

All infection assays were carried out as described previously [29]. The L4 stage of nematodes was exposed to *S. sonnei* and *S. flexneri* lawns. One hundred worms were harvested at two time points, 10 min (10 mpi) and 24 h post-infection (24 hpi). Tri-RNA (Favorgen), in combination with the Monarch Total RNA Miniprep kit (New England Biolabs, Massachusetts, USA), was used according to the manufacturer’s instructions to purify the RNA. *In vitro S. sonnei* 29930 cells grown on NGM agar overnight were also harvested for RNA extraction.

### Transcriptome bioinformatic analyses

All RNA library samples were prepared as described [29] and sequenced on the Illumina Novaseq 6000 system (150 bp paired-end). The raw reads were trimmed using Trimmomatic v0.39 [30] and mapped to the *C. elegans* genome (NCBI RefSeq assembly GCF_000002985.6) using hisat2 v2.2.1 [31]. The unmapped reads were then mapped to *S. sonnei* ATCC 29930 or *S. flexneri* ATCC 12022 genome obtained from the ATCC Genome Portal [32]. The mapped reads were quantified using htseq-count [33]. The differentially expressed genes (DEGs) were determined with DESeq2 [34], using a threshold of FDR<0.05 and a log2fold change of ±1. Single orthologs between the two *Shigella* strains were obtained using ProteinOrtho [35], with the functional nucleotide sequences as input. Functional enrichment was performed using the online tool ShinyGo v0.80 [36].

### Knockout mutants of *S. sonnei* ATCC 29930

Knockout mutants of the target genes in the *S. sonnei* ATCC 29930 wild type (WT) were constructed using homologous recombination as previously described [37] using the chloramphenicol resistance cassette as a selection marker. These genes are *cspD* and hok toxin genes. After electroporation, the bacteria were plated on agar containing 15 µg ml^−1^ of chloramphenicol for selection. Successful knockout mutants were verified by colony PCR.

### Growth curve of mutants

The growth curves of the *S. sonnei* mutants and WT were obtained and compared. The overnight cultures of the bacteria were diluted to 0.05 OD and added to the microplate. The plate was incubated at 37 °C, and the OD 600 nm reading was obtained every 30 min. The maximum specific growth rate was determined using a growth curve fitting the Gompertz model, and the results from five replicates were analysed for any statistical significance using one-way ANOVA with the nonparametric Kruskal–Wallis test and Dunn’s multiple comparison tests.

### Killing assay and bacterial colonization assay of nematodes

The virulence of the *S. sonnei* mutants was determined using the nematode killing assay and bacterial colonization assay as described previously [20,29, 38].

## Results and discussion

### Dual RNA sequencing of the *Shigella-*infected *C. elegans*

**Fig. 1.**
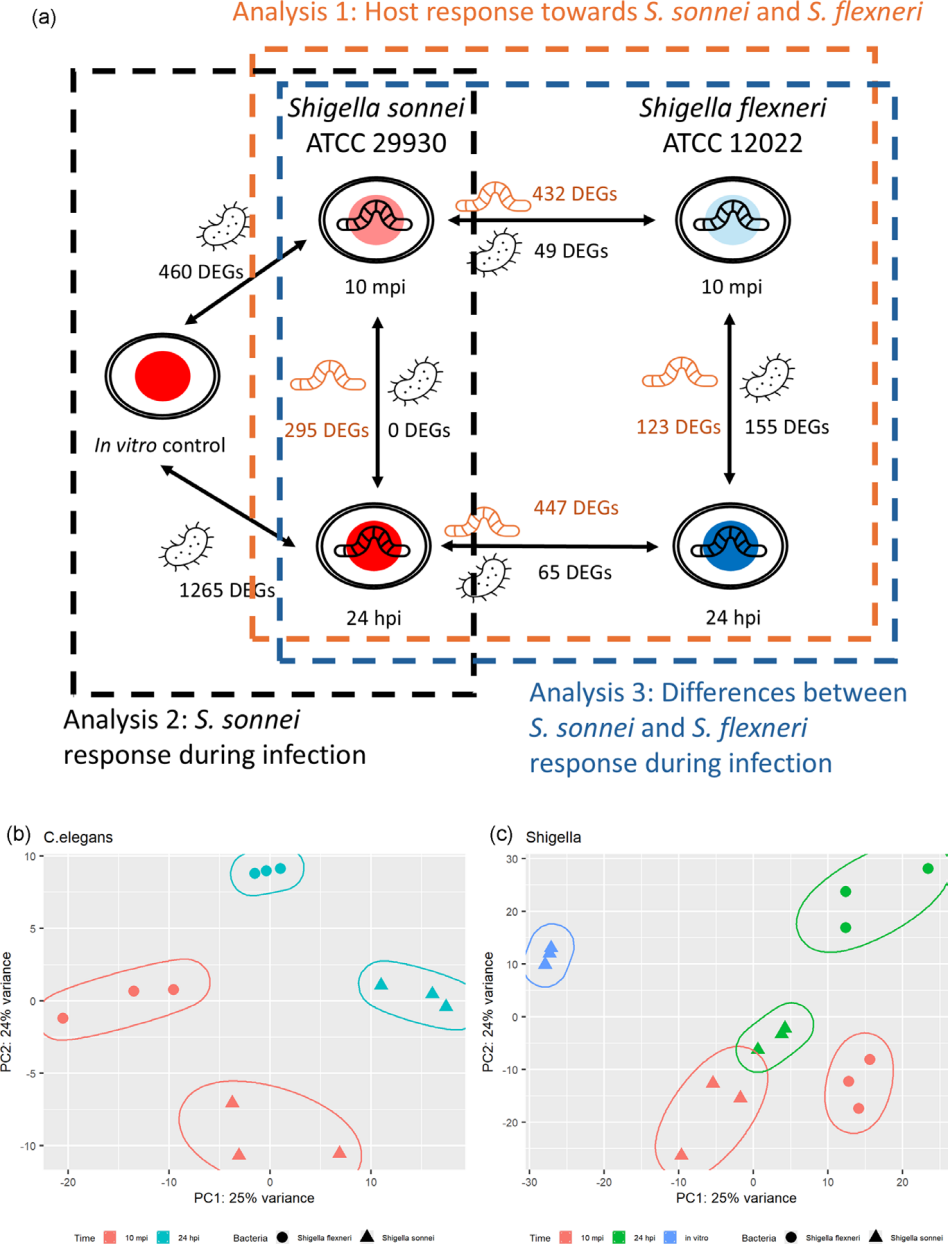
*C. elegans–Shigella* host–pathogen infection samples. (a) A total of five conditions were used to collect RNA for sequencing (*S. sonnei in vitro*, *S. sonnei*-infected *C. elegans* 10 mpi, *S. sonnei-*infected *C. elegans* 24 hpi, *S. flexneri-*infected *C. elegans* 10 mpi and *S. flexneri-*infected *C. elegans* 24 hpi). The number of differentially expressed genes (DEGs) (*P*-value<0.05, fold change >2) are labelled between the conditions compared (orange indicates DEGs by *C. elegans*; black indicates DEGs by *Shigella*). Boxes indicate the samples used in the three different parts of the analysis. (**b)** The principal component analysis (PCA) plot of *C. elegans* during *S. sonnei* or *S. flexneri* infection at 10 mpi and 24 hpi. (**c)** PCA plot of the *S. sonnei* or *S. flexneri* response during *C. elegans* infection at 10 mpi and 24 hpi.

*S. sonnei* ATCC 29930 and *S. flexneri* ATCC 12022 do not contain the invasion plasmid required for virulence. However, the nematodes infected with these two *Shigella* had a significant decrease in lifespan compared to the non-pathogenic *E. coli* [29]. Additionally, there is a higher amount of *Shigella* obtained from the nematode gut after infection than *E. coli* OP50. A previous study had only investigated the bacterial response during *C. elegans* infection at 72 hpi. To further investigate the bacterial and host transcriptional response during infection, the total RNA from infected *C. elegans* at two time points (10 mpi and 24 hpi) was sequenced (Fig. 1). The reads were first mapped to *C. elegans*. Next, the unmapped reads were mapped to either *S. sonnei* or *S. flexneri*. An average of 66M reads was obtained from the *S. sonnei*–*C. elegans* samples, while 105M reads were obtained from the *S. flexneri*–*C. elegans* samples. More than 83% of reads remained after trimming and quality filtering, while the remaining reads did not pass quality filtering. For dual RNA-sequencing samples, more than 98% of the reads mapped to the *C. elegans* genome, while 0.02–0.47% of the reads mapped to the bacterial genome (Table S1, available in the online version of this article). The low amount of bacterial reads was expected due to the different genome sizes between the host and the bacteria [39]. All samples from the same conditions were clustered in the principal component assay plot (Fig. 1b, c). However, *S. sonnei* response at both time points was clustered closer to each other than *S. flexneri* (Fig. 1c), suggesting that their response at 24 hpi is similar to the beginning of infection.

### *C. elegans* has different responses towards *S. sonnei* or *S. flexneri* infection

**Fig. 2.**
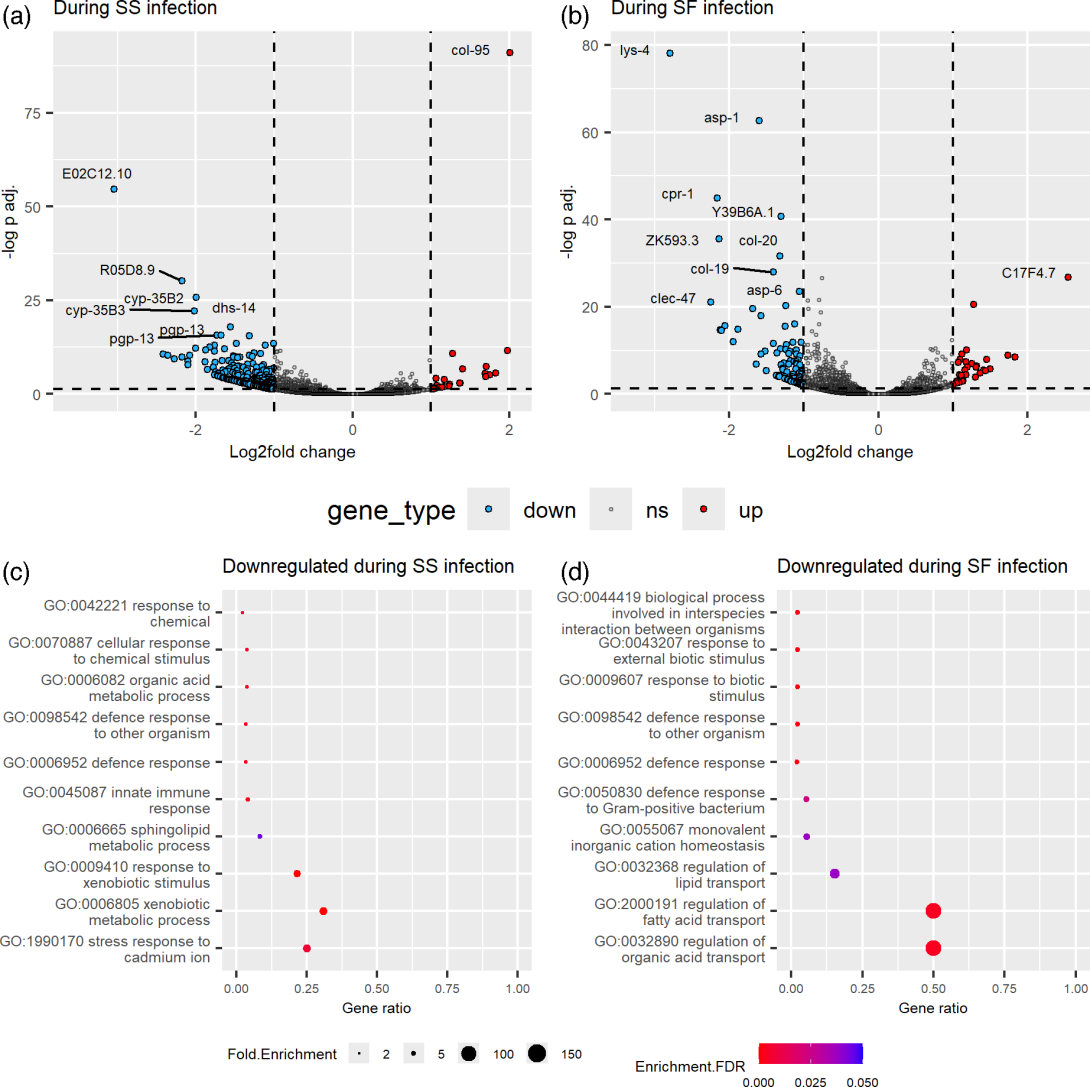
*C. elegans* response during *S. sonnei* or *S. flexneri* infection at 10 mpi and 24 hpi. (a) Volcano plot of DEGs in *C. elegans* during *S. sonnei* infection. (**b)** Volcano plot of DEGs in *C. elegans* during *S. flexneri* infection.** (c)** The enriched gene ontology (GO) biological processes identified among the downregulated DEGs in *S. sonnei-*infected nematodes. (**d)** The enriched GO biological processes identified among the downregulated DEGs in *S. flexneri-*infected nematodes.

*S. sonnei*-infected *C. elegans* differentially regulates 295 genes at the later infection stage (Fig. 2a), while *S. flexneri*-infected *C. elegans* differentially regulates 123 genes (Fig. 2b; DEGs listed in Tables S2 and S3, available in the online version of this article). Multiple pathways were enriched within the downregulated genes in *C. elegans* during the infection of *S. sonnei* (Fig. 2c) and *S. flexneri* (Fig. 2d). First, the infection of both *S. sonnei* and *S. flexneri* led to the downregulation of several genes involved in *C. elegans* defence response at 24 hpi. *S. sonnei*-infected *C. elegans* also downregulate genes involved in the stress response to cadmium ion (*cdr-1*, *cdr-4* and *pgp-3*), response to xenobiotic stimulus (*cyp-33C1*, *cyp-33C7*, *cyp-34A10*, *cyp-34A7* and nine *cyp-35* genes) and sphingolipid metabolic process (*asah-1*, *asah-2*, *asm-2*, *cgt-1* and *gba-2*). In contrast, *S. flexneri*-infected *C. elegans* downregulates genes involved in monovalent inorganic cation homeostasis (*amt-1*, *nhx-2* and *pept-1*).

Similar to this study, the nematode defence response is commonly enriched during bacterial infection [40]. For instance, the defence response genes were upregulated by the *S. flexneri*-infected nematodes compared to non-infected nematodes at the same time of infection [27]. However, our study demonstrated that the defence response was downregulated as the infection progressed from early to later stages. The downregulation of the host defence response, or host immune suppression, can be induced by the bacteria as a part of their pathogenesis repertoire, including *Shigella* [41,43]. However, these are mainly mediated by the plasmid-encoded T3SS [44,45]. As the studied *Shigella* strains do not have the pINV plasmid, this suggests that *S. sonnei* and *S. flexneri* may be suppressing these nematode defence response genes with other mechanisms.

During *S. sonnei* infection, *C. elegans* downregulated genes related to its xenobiotic response. The *C. elegans* xenobiotic response has been shown to interact with the immune response towards bacterial infection [46]. However, their specific role during *S. sonnei* infection is unclear. These genes may be induced at the early stages of infection and then downregulated after they are no longer needed. In addition, these genes are only downregulated in *C. elegans* during *S. sonnei* infection, suggesting that these may be responses against *S. sonnei* specifically. In contrast, *S. flexneri*-infected nematodes downregulate monovalent inorganic cation homeostasis genes, which are *pept-1* and *nhx-2*. The peptide transporter PEPT-1 regulates the uptake of free fatty acids along with the sodium-proton exchanger NHX-2, and PEPT-1 loss leads to higher free fatty acid uptake [47]. While these proteins were not directly linked to the nematode response towards *Shigella* or any bacterial infection, they were downregulated in this study. This demonstrated that there are differences in *C. elegans* response towards *S. sonnei* or *S. flexneri* infection, which is similarly reported in other studies [48,49].

### *S. sonnei* upregulates biofilm formation and energy generation/conservation during *in vivo* growth in *C. elegans*

**Fig. 3.**
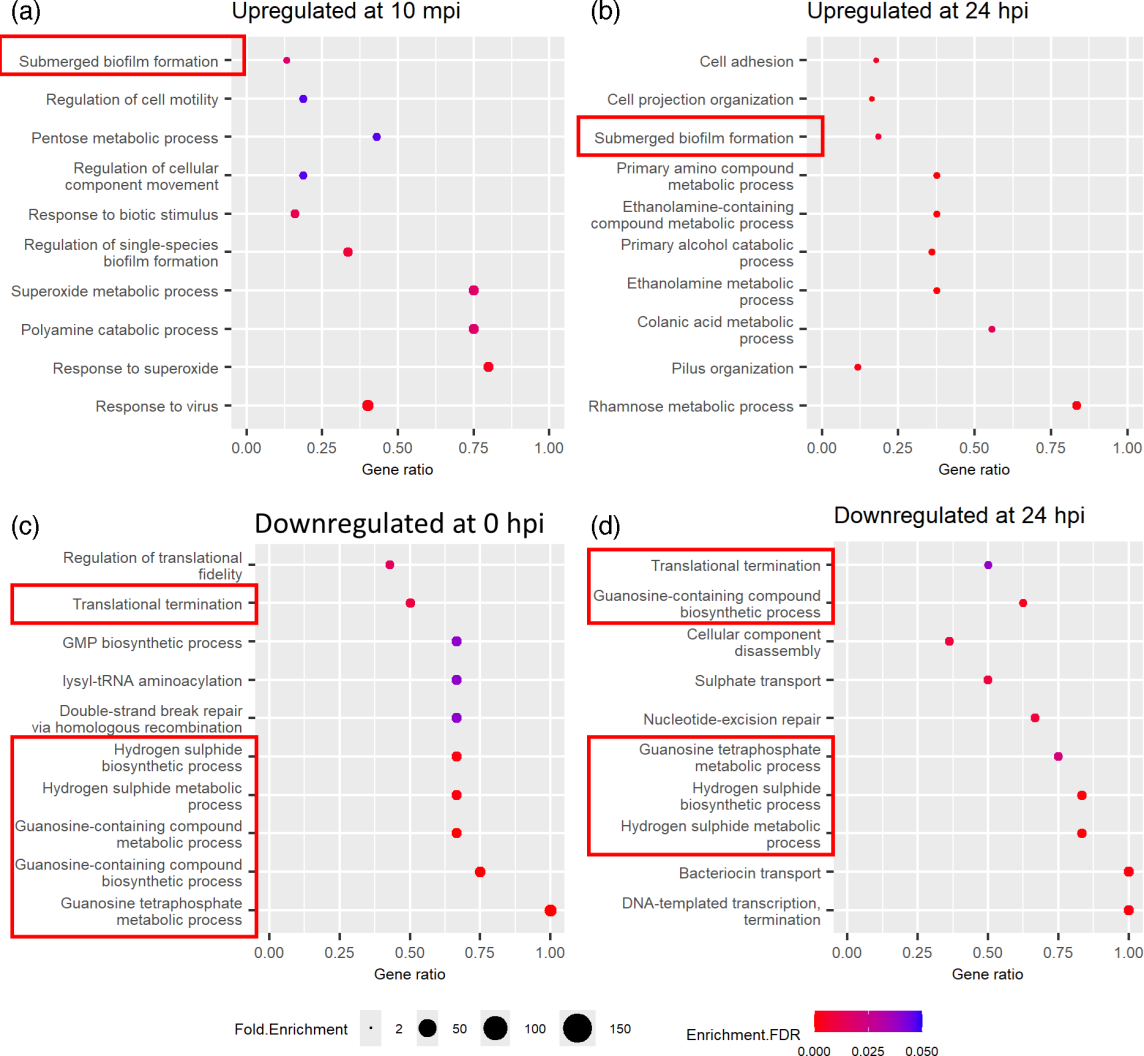
Enriched GO biological processes identified among the DEGs in *S. sonnei* during *in vivo C. elegans* infection at 10 mpi and 24 hpi. (a) Enriched within DEGs upregulated at 10 mpi. (**b)** Enriched within DEGs upregulated at 24 hpi. (**c)** Enriched within DEGs downregulated at 10 mpi. (**d)** Enriched within DEGs downregulated at 24 hpi. Red boxes indicate the common pathway enriched between two time points of infection.

There are 460 *S. sonnei* genes differentially regulated after 10 min of infection, while 1265 genes were differentially regulated after 24 h of infection (DEGs listed in Tables S4 and S5). Functional enrichment analysis was performed on these genes to obtain the top ten pathways with the highest fold enrichment, and multiple pathways were commonly enriched at both time points of infection (Fig. 3). The submerged biofilm formation genes (*ybgD*, *bssS*, *ychH* and *yfcV* genes) are commonly upregulated at both time points (Fig. 3a, b). In contrast, the translation termination genes (*frr*, *prfA* and *prfB*), guanosine-containing compound biosynthetic process genes (*guaA*, *guaB*, *relA*, *spot* and *gppA*) and hydrogen sulphide metabolic process genes (*cysD*, *cysI*, *cysJ* and *cysN*) were downregulated by *S. sonnei* at both time points (Fig. 3c, d).

Interestingly, an intermembrane transport protein gene, *yebS*, is upregulated at 10 mpi but downregulated at 24 hpi (which appears bold in Table S4 and S5). This gene contributes to membrane integrity in *E. coli*. However, its exact function is unknown [50]. As YebS has not been linked with bacterial pathogenesis before, its role in *S. sonnei* at early and later stages of *C. elegans* infection is currently unknown.

Biofilm formation genes were upregulated during both time points of infection (Fig. 3a, b). Biofilm formation is associated with host immune system evasion and survival during adverse conditions such as low pH in the gut [51]. Biofilm is also induced in *S. flexneri* by bile salts, indicating its role during gut infection [52,55]. Other related genes, such as cell motility and biofilm regulatory genes, are also upregulated at 10 mpi, including *bssR* and *bssS* [56], *adrA* [57], *glgS* [58] and *uspG* [59], suggesting that fine-tuning biofilm formation during the early *C. elegans* infection is crucial for *S. sonnei*. Additionally, *S. sonnei* upregulates genes in pilus organization, cell projection organization and cell adhesion at 24 hpi, including multiple flagellar genes such as *flg* and *fli* genes. As biofilm formation requires cell aggregation and adhesion, cell projections such as pili and flagella help support the initial interactions between bacterial cells to form microcolonies [60,61]. Thus, even though *Shigella* do not have functional flagella [62], the upregulation of biofilm regulatory genes may indirectly lead to increased flagellar gene expression.

*S. sonnei* also prioritizes energy generation or conservation during *in vivo* infection (Fig. 3). *S. sonnei* upregulates catabolic processes and energy derivation/generation and downregulates biosynthesis and DNA repair/replication during *C. elegans* infection. This includes the upregulation of polyamine catabolism at 10 mpi and the upregulation of rhamnose, colanic acid and other general catabolism at 24 hpi. *S. sonnei* also downregulates genes involved in hydrogen sulphide and guanosine-containing compound metabolism, sulphate and protein transport, as well as DNA replication and repair during *C. elegans* infection. Additionally, carbohydrate transport was upregulated at 24 hpi, providing nutrients and supporting bacterial growth in the host through increased glucose uptake [63]. Due to multiple challenges during *C. elegans* gut infection, such as insufficient nutrients, low pH and the host’s immune system [64], the generation and conservation of energy can support *S. sonnei*’s continued survival.

### The membrane lipid metabolism is differentially regulated by both *C. elegans* and *S. sonnei* at 24 hpi simultaneously

The dual RNA sequencing and analysis allow the simultaneous observation of the host and pathogen response simultaneously. In this study, *C. elegans* downregulates sphingolipid metabolism genes (*asah-1* and *asah-2*) (Fig. 2c), while *S. sonnei* upregulates ethanolamine metabolism genes (*ccmL*, *eutE*, *eutN*, *eutP*, *eutQ*, *eutS*, *eutT*, *pduB* and *pudU*) at 24 hpi (Fig. 3b). The simultaneous regulation of the membrane lipid metabolism in *C. elegans* and *S. sonnei* during 24 h of infection demonstrated that these membrane lipids have roles in both host defence and bacterial pathogenesis. Interestingly, the sphingolipid metabolism genes were not differentially regulated in *S. flexneri*-infected nematodes.

Sphingolipids are one of the membrane lipids in eukaryotes, and they have multiple roles in bacterial infection [65]. Both *asah-1* and *asah-2* genes are involved in sphingolipid catabolism, and the downregulation of sphingolipid catabolism improves host survival during bacterial infection [66]. Other sphingolipid metabolism genes provide protective activity towards *P. aeruginosa* and *Enterococcus faecalis* [67]. Thus, the downregulation of these genes in the nematodes suggests that this may be one of the protective responses against *S. sonnei* infection, but not *S. flexneri*, at 24 hpi.

Bacteria can either synthesize or exploit the host’s sphingolipids for pathogenesis [68]. *Shigella* relies on the host sphingolipids for pathogenesis, as *S. flexneri* invasion is reduced in host cells that are sphingolipid-deficient [69]. Phosphatidyl-ethanolamine, a precursor membrane lipid and a byproduct of sphingolipid catabolism, is important for host cell attachment [70,71]. *E. coli* has been shown to alter the host phospholipid metabolism during infection [72], suggesting that the upregulation of ethanolamine metabolism by *S. sonnei* at 24 hpi is most likely helpful for pathogenesis.

### *S. sonnei* upregulates acid resistance (AR) and biofilm genes compared to *S. flexneri* during *C. elegans* infection

**Fig. 4.**
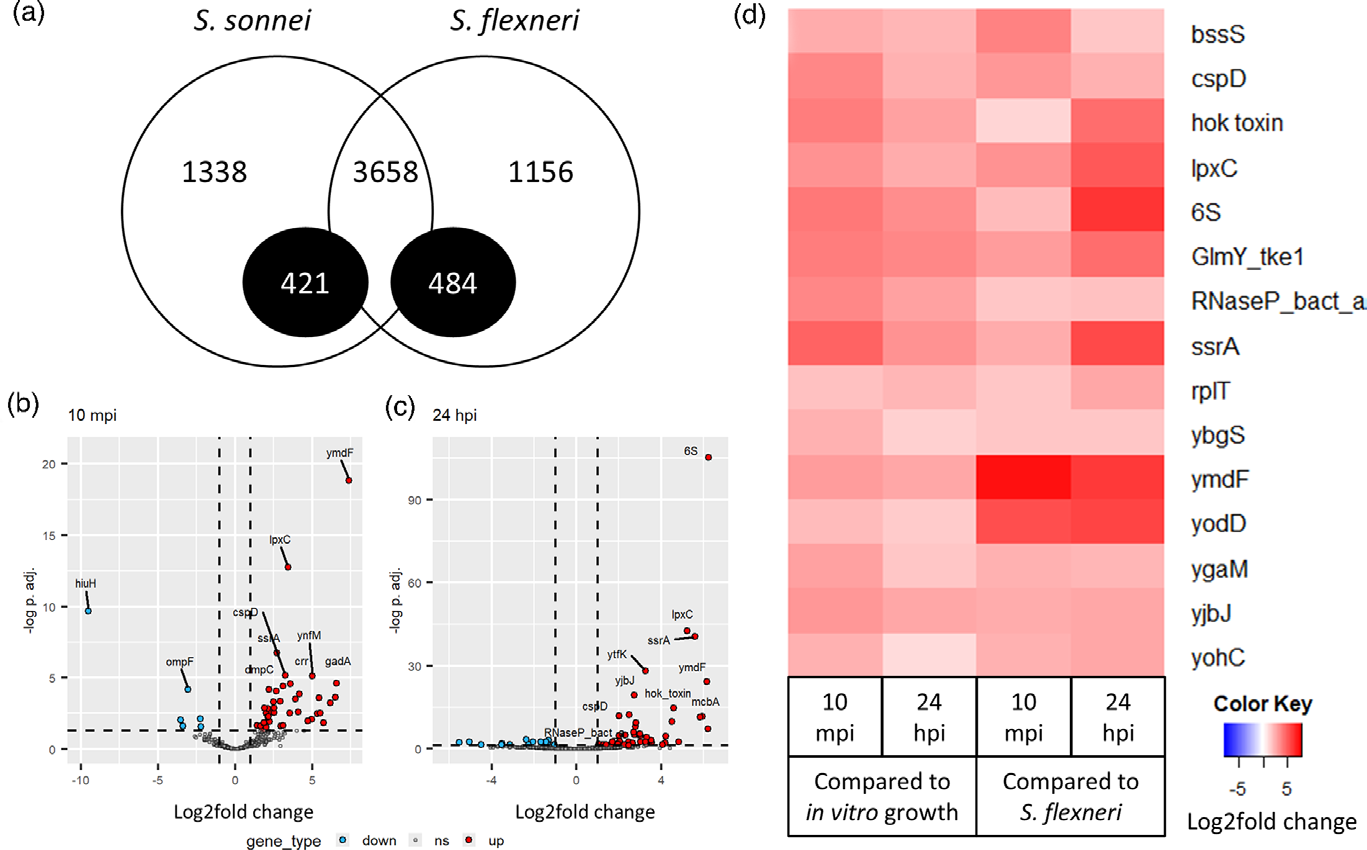
Genomic and transcriptomic differences between *S. sonnei* ATCC 29930 and *S. flexneri* ATCC 12022 during *C. elegans* infection. (a) The number of orthologous genes between *S. sonnei* and *S. flexneri was* identified using ProteinOrtho. Black circles denote the genes with multiple orthologs in either strain, which are mostly hypothetical proteins. (b, c) Volcano plots of DEGs between *S. sonnei* and *S. flexneri* during (**b)** 10 mpi and (**c)** 24 hpi of *C. elegans* infection. (**d)** Heatmap of the 15 DEGs commonly identified from both analyses.

*S. sonnei* and *S. flexneri* have 5417 and 5298 total annotated genes, respectively. There are 3658 genes with a single ortholog in each bacterial genome, while other genes were either specific to each strain or have multiple orthologs (Fig. 4a). A high number of these genes are hypothetical proteins. For the direct comparison of the transcriptomic responses of *S. sonnei* and *S. flexneri* during *C. elegans* infection, only the 3658 common genes were analysed. Between the responses of *S. sonnei* and *S. flexneri* during infection, 49 genes were differentially regulated at 10 mpi, while 65 genes were differentially regulated at 24 hpi (Fig. 4b, c; DEGs listed in Tables S6 and S7). At 10 mpi, *S. sonnei* upregulates genes involved in AR compared to *S. flexneri*, which are genes involved in the glutamate-dependent system (*gadA*, *gadB* and *gadC*) and the acid stress chaperones (*hdeA* and *hdeB*). At 24 hpi, *S. sonnei* also upregulates biofilm-related genes, including the two biofilm regulatory genes *bssR* and *bssS*.

*Shigella* can persist in the acidic gut environment due to their glutamate decarboxylase-dependent AR system. The proteins GadA, GadB and GadC and the acid chaperones HdeAB play key roles in maintaining the intracellular pH at near-neutral conditions [73,74]. This study suggested that *S. sonnei* upregulates its AR mechanism at the early stages of *C. elegans* infection, perhaps responding to the low pH environment earlier than *S. flexneri*. Additionally, biofilm is also involved in *Shigella* response towards the gut conditions, as biofilm formation can be triggered by bile acid in *S. flexneri* [53]. Most studies on AR [73,75,78] and biofilm [79,80] were performed on *S. flexneri*, while none were performed on *S. sonnei*. The upregulation of AR and biofilm in *S. sonnei* provides possible explanations for the differences in virulence between *S. sonnei* and *S. flexneri*. However, further investigation is required to elucidate the differences between these two species.

### Knocking out CspD and a hok toxin in *S. sonnei* did not negatively affect the virulence of *S. sonnei*

There are 15 common genes upregulated during *S. sonnei* infection and expressed at significantly higher levels in *S. sonnei* than in *S. flexneri* (Fig. 4d). Two genes (*cspD* and hok toxin gene) were selected for further validation using gene knockout. The CspD protein is linked with persister formation, which protects the bacteria from host immune response [81]. The chromosome-encoded hok toxin prolongs the lag phase of bacteria to allow time for its stress response [82]. While deleting these genes did not affect their *in vitro* growth, there were also no differences in their virulence during *C. elegans* infection compared to the WT (Fig. S1). This suggests that while the hok toxin gene and *cspD* genes are differentially regulated within *S. sonnei* during the infection of *C. elegans* at 24 hpi, single gene deletion may not be sufficient to reduce the virulence of *S. sonnei* for a longer period.

### Limitation of study

This study has multiple limitations. First, *C. elegans* is a soil nematode with an optimal temperature lower (20–25 °C) than the human body temperature (37 °C). Thus, the infection was performed at a lower temperature than is physiologically relevant. Nevertheless, as the nematodes demonstrated a significant decrease in lifespan after *Shigella* infection, this study provided some preliminary data on the *Shigella–C. elegans* infection model. Next, the number of reads that are mapped to the *Shigella* genome is relatively low compared to other bacterial transcriptomic studies. Thus, this study can only capture highly expressed bacterial genes. Nevertheless, this study provided some pathways and targets of interest involved in *Shigella* infection within *C. elegans* gut that are significantly differentially expressed during the infection.

## Conclusion

In conclusion, this study provided insights into the response of *C. elegans*, *S. sonnei* and *S. flexneri* during 10 mpi and 24 hpi. *C. elegans* downregulated its defence response at a later stage of *Shigella* infection. *S. sonnei-*infected nematodes also downregulated their membrane lipid sphingolipid metabolism. *S. sonnei* also upregulates the membrane lipid precursor ethanolamine metabolism. *S. sonnei* also upregulates biofilm formation and prioritizes energy generation/conservation during *C. elegans* infection. Finally, the two *Shigella* species have slight transcriptomic response differences during *C. elegans* infection, as *S. sonnei* significantly upregulates its biofilm- and acid-resistance-related genes compared to *S. flexneri*. This study demonstrated that *S. sonnei* and *S. flexneri* have species-specific responses during pathogenesis that may affect their virulence.

## supplementary material

10.1099/mgen.0.001339 Uncited Supplementary Material 1.

10.1099/mgen.0.001339 Uncited Supplementary Material 2.

## Abbreviations

AR: acid resistance
ATCC: American Type Culture Collection
DEGs: differentially expressed genes
GO: gene ontology
hpi: hours post-infection
mpi: minutes post-infection
NGM: nematode growth medium
PCA: principal component analysis
T3SS: type III secretion system
WT: wild type
