# Supplementary material for: Shigella sonnei and Shigella flexneri infection in Caenorhabditis elegans led to species-specific regulatory responses in the host and pathogen
Source: Microb Genom. 2025 Jan 24;11(1):001339. doi: 10.1099/mgen.0.001339 (PMC11893279; doi:10.1099/mgen.0.001339)
Supplement: Uncited Supplementary Material 1. [file mgen-11-01339-s001.pdf]

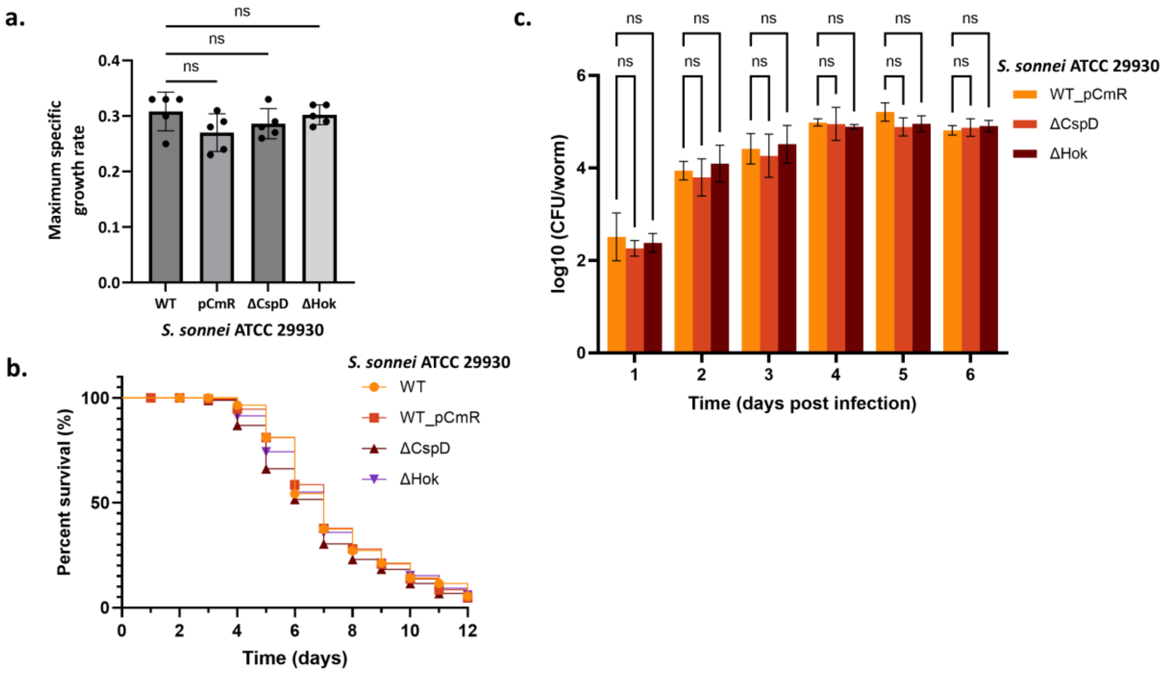

Supplementary Figure 1: Characterization of the *S. sonnei* ATCC 29930 wild type (WT), WT with pUltra-CmR plasmid, and the two knockout mutants (ΔCspD and ΔHok). **a.** The maximum specific growth rate of the strains grown in LB broth. **b.** Killing assay of *C. elegans* by the WT and the mutant strains (n = 150 worms). **c.** Bacterial colonisation assay of *C. elegans* infected with the WT-pUltra-CmR (for antibiotic selection) and mutant strains (n = 30 worms). ns indicate statistically non-significant results in Kruskal-Wallis ANOVA test (a), log-rank (Mantel-Cox) test (b) and two-way ANOVA with Sidak multiple comparison tests (c).
